# Sakuranin represses the malignant biological behaviors of human bladder cancer cells by triggering autophagy via activating the p53/mTOR pathway

**DOI:** 10.1186/s12894-023-01334-2

**Published:** 2023-10-24

**Authors:** Ling Hao, Dandan Mu, Haitao Mu

**Affiliations:** 1 Department of Medical Oncology, The Fourth Affiliated Hospital of Harbin Medical University, No.37, Yiyuan Street, Harbin, 150000 China; 2 Department of Medical Oncology, The Second Affiliated Hospital of Harbin Medical University, Harbin, China; 3 Department of Medical Oncology, The Fifth Hospital of Harbin, Harbin, China

**Keywords:** Sakuranin, Bladder cancer, T24 cells, Autophagy, p53/mTOR pathway, Proliferation, Apoptosis

## Abstract

**Objective:**

Sakura extract is a natural flavonoid compound that may have potential anti-tumor effects. The paper focuses on investigating Sakuranin mechanism on bladder cancer (BC) cells.

**Methods:**

BC cells (T24) were treated with different concentrations of Sakuranin, with 48-h IC50 determined. T24 cells were treated with Sakuranin at IC50, followed by assessment of cell proliferative/apoptotic/migrative/invasive activities by CCK-8, EdU and plate clone formation assays/flow cytometry/Transwell/scratch test. MMP-2 (migration and invasion-related protein) protein level was assessed by Western blot. Cell autophagy was evaluated by measuring the protein levels of autophagy markers (LC3-I/LC3-II/p62) through Western blot. The autophagy inhibitor 3-MA was used to validate the role of autophagy in the regulatory mechanism of Sakuranin in T24 cell behaviors. Furthermore, the activation of the p53/mTOR pathway in cells was detected and a combination of Sakuranin and p53 inhibitor Pifithrin-µ was adopted to explore the involvement of this pathway.

**Results:**

Sakuranin decreased T24 cell proliferation/EdU positive cell percentage/colony formation number and area/migration/invasion/scratch healing/MMP-2 protein level, and accelerated apoptosis. Sakuranin elevated the LC3-II/I ratio and lowered p62 level in T24 cells. 3-MA partially averted Sakuranin-mediated repression on cell malignant behaviors. Sakuranin upregulated p-p53 and p53 levels, and decreased the p-mTOR/mTOR ratio in T24 cells. The effects of Sakuranin on cell biological behaviors were partly annulled by Pifithrin-µ treatment.

**Conclusion:**

Sakuranin suppressed T24 cell proliferation/migration/invasion, and enhanced apoptosis by potentiating autophagy through activating the p53/mTOR pathway. This study provided a theoretical basis for Sakuranin as a potential drug for clinical treatment of BC.

**Supplementary Information:**

The online version contains supplementary material available at 10.1186/s12894-023-01334-2.

## Introduction

Bladder cancer (BC) is conceived as the most-frequently diagnosed malignancy of the urinary tract and is chiefly characterized by histologic and molecular heterogeneity [1, 2]. As indicated by global cancer statistics, BC is responsible for 573,278 new cases and 212,536 cancer-associated deaths in 2020, which has been emerged as the 10th most prevalent malignancy globally [3]. Urothelial carcinoma represents the most universal histologic type, whereas small cell carcinoma, squamous cell carcinoma, sarcoma, and adenocarcinoma are rarer [4]. A majority of patients (70%) present with non-muscle-invasive BC (NMIBC) at diagnosis, which has a high risk of recurrence (50-70%) and easily progresses to MIBC in approximately 10–20% cases [5]. Currently, therapeutic modalities for BC generally consist of surgery, chemotherapy, radiotherapy, and immunotherapy [6]. However, recurrence and metastasis rates of BC are still high, and treatment agents are associated with several limitations, such as side effects, poor solubility, low bioavailability, and tumor resistance [7, 8]. This reality underscores the unmet need to find more efficacious and novel treatments for BC to ultimately prolong overall survival and improve treatment outcomes of patients.

Phytochemicals are generally considered to be natural compounds in plants as secondary metabolites, which are currently exploited for developing novel therapeutic strategies against cancer due to their essential functions in modulating the levels of transcripts with oncogenic or tumor-suppressing roles [9]. Amongst these, flavonoids are a dominant class of natural products widely present in plants and primarily known for their cardio-protective, anti-oxidant, anti-inflammatory, anti-mutagenic, and anti-cancer properties, the activity of which depends largely on the structure as well as the degree of oxidation, unsaturation, and polymerization of the “C” ring [10, 11]. More importantly, flavonoids bring less psychological stress and financial burden to patients due to their harmlessness and wide availability [12]. To our knowledge, Sakuranetin is recently identified as an imperative plant flavonoid with anti-cancer, anti-protozoal, anti-microbial, and anti-viral properties, and its glycoside, termed Sakuranin, is first isolated from the bark of *Prunus pseudo-cerasus* [13]. Therefore, we speculated that Sakuranin may also have a therapeutic effect on BC and meanwhile investigated the possible mechanism.

p53, encoded by the *TP53* gene, is broadly acknowledged as a tumor-suppressor and its activation in response to various stresses is essential for the survival of normal cells and protection of these cells against tumorigenesis [14]. Mutation in the *TP53* gene occurs in approximately 50% of human cancers, including bladder, lung, liver, and prostate cancers [15]. Of note, p53 confers vital roles in multiple biological processes, such as DNA repair, antioxidant defense, cell metabolism, apoptosis, senescence, and cell cycle arrest, contributing to the functions of p53 in tumor suppression [16]. Interestingly, several flavonoids have been documented to be related to p53 activation in BC: for instance, either galangin or fisetin is capable of inducing BC cell apoptosis via activation of the p53 signaling [17, 18]. Hence, it seems reasonable to speculate that Sakuranin may similarly exert a regulatory effect on p53. Furthermore, p53 is reported to modulate the complicated machinery of the mammalian target of rapamycin (mTOR) pathway at diverse levels to manipulate cellular processes, such as cell apoptosis, proliferation, autophagy, migration, and tumorigenesis [19]. In light of the above background, we intended to investigate the action of Sakuranin in modulating BC biological behaviors through the p53/mTOR pathway.

## Materials and methods

### Cell culture

Human BC cell line T24 (HTB-4, ATCC, Manassas, VA, USA) was cultured in RPMI-1640 medium (Gibco, Gaithersburg, MD, USA) comprising 10% fetal bovine serum (FBS) and penicillin (100 U/mL)-streptomycin (100 mg/mL) double antibodies at 37 °C with 5% CO_2_. After growing to the logarithmic phase, cells were detached with 0.25% trypsin digestion solution (SH-2464, Kaishiyuan Biotechnology, Beijing, China) and passaged once every 2–3 days. After 3 passages, T24 cells at logarithmic phases were collected for subsequent experimentation.

### Toxicity test of sakuranin

T24 cells (5 × 10^3^ cells/well) were put on 96-well plates for adhesion and incubated with Sakuranin at concentrations of 0, 1.25, 2.5, 5, 10, 20, 40 and 80 mg/mL (CAS No.: 529-39-5, Shishun Biotechnology, Tianmen, Hubei, China), with sterile water as the solvent of Sakuranin. Cell viability was detected using cell counting kit-8 (CCK-8) kits (A311-01, Vazyme, Nanjing, Jiangsu, China) at 24, 48 and 72 h. The optical density of each well at 450 nm (OD450 value) was measured utilizing a microplate reader (Bio-Rad, Hercules, CA, USA). Thereafter, the 24-h, 48-h and 72-h half-maximal inhibitory concentration (IC50) of Sakuranin on T24 cells was calculated by GraphPad Prism 8.0 (GraphPad Software Inc., San Diego, CA, USA), and subsequent studies were performed with the IC50 of 48 h referring to the exsiting study [20], and considering the results that T24 cells showed the highest sensitivity at 48 h.

### Cell grouping and treatment

T24 cells were placed onto 96-well plates at 5 × 10^3^ cells/well and grouped as follows: (1) blank group; (2) Sakuranin group: treated with Sakuranin at IC50 concentration (6.8 mg/mL) for 48 h; (3) Sakuranin + 3-MA group: treated with autophagy inhibitor 3-MA (2 mM, HY-19,312, MedChemExpress, Princeton, NJ, USA) for 2 h, followed by 48-h treatment with Sakuranin. Sterile water was adopted as solvent of 3-MA, and the dosage of 3-MA was referred to prior research [21]; (4) Sakuranin + DMSO group and (5) Sakuranin + Pifithrin-µ group: treated for 2 h with dimethyl sulphoxide (DMSO) or Pifithrin-µ (2 µM, HY-10,940, MedChemExpress), followed by 48-h treatment with Sakuranin. Pifithrin-µ was dissolved in DMSO, the dosage of which was based on previous studies [20].

### CCK-8 assay

The evaluation of cell viability was performed using CCK-8 kits (A311-01, Vazyme). T24 cells were put on 96-well plates and subjected to different treatments. After 24, 48 and 72 h of treatment, each well was supplemented with CCK-8 solution and then its OD450 value was detected by a microplate reader (Bio-Rad, Hercules, CA, USA).

### EdU assay

Cell proliferation was assessed using the EdU cell proliferation detection kit (C0071S, Beyotime, Shanghai, China). Differently-treated cells (5000 cells/well) were cultured in 96-well plates for 24 h, supplemented with a final concentration of 50 µmol/L EdU solution per well, and incubated at 37 °C for 2 h. Then, cells were fixed at room temperature with 4% paraformaldehyde for 15 min, incubated in the dark with Apollo fluorescent staining solution for 30 min, washed twice with methanol, and stained with DAPI staining solution for 30 min at room temperature, followed by three rinses with phosphate-buffered saline (PBS). The total number of cells and the number of EdU positive cells from 5 fields randomly selected under a fluorescence microscope (Leica, Germany) were applied for calculation of the percentage (%) of EdU positive cells.

### Plate clone formation assay

Cell growth ability was assessed by clone formation assay. T24 cells were treated with Sakuranin for 48 h, detached into cell suspension using trypsin, and seeded into 6-well plates at a count of 1000. After incubation for 2 weeks in the complete medium, cells were rinsed thrice with PBS, fixed with methanol at room temperature for 30 min, and then stained with 0.5% crystal violet (G1065, Solarbio, Beijing, China) for 5 min, and imaged under a microscope (Olympus, Tokyo, Japan). The average area (cm^2^) of clones was analyzed using image processing software Image Pro Plus 6.0 (Media Cybernetics, Silver Spring, MD, USA).

### Flow cytometry

Cell apoptosis was evaluated with Annexin V-fluorescein isothiocyanate (FITC)/propidium iodide (PI) Apoptosis Detection Kit (A211-01, Vazyme). T24 cells post different treatments were collected and next centrifuged (120×g, 5 min) at room temperature, with the supernatant discarded. Following washing once with PBS, cells were subjected to fixing for 12 h with 70% ethanol that was pre-chilled at -20 °C, centrifugation (120×g, 5 min) at room temperature, and rinsing once with PBS. Cells were subsequently added with 5 µL PI and 10 µL Annexin V-FITC solution and mixed well, followed by placing for 30 min at room temperature under conditions devoid of light. The apoptosis was detected utilizing a flow cytometer (Becton Dickinson, San Jose, CA, USA).

### Transwell assay

Transwell assays were performed for detection of T24 cell migration and invasion. Transwell chambers of 8-µm pore size (Corning, Corning, NY, USA) were placed in 24-well plates, and the apical chamber was covered (to detect invasion) or not added (to detect migration) with 60 µL Matrigel (Becton Dickinson). T24 cells (approximately 1 × 10^4^ cells) were added to the apical chamber, and 800 µL complete medium containing 10% FBS was supplemented to the basolateral chamber. After incubation at room temperature for 24 h, the chambers were removed, and the non-migrating or non-invading cells in the apical chamber were wiped off with cotton swabs. The T24 cells migrated or invased into the basolateral chamber were fixed for 30 min in 100% methanol and stained for 30 min with 0.1% crystal violet. Later, 5 non-overlapping fields were arbitrarily selected under a microscope (Olympus) for observing and taking pictures. The number of T24 cells that migrated or invaded was counted. The experiment was repeated 3 times, and the average was taken.

### Scratch test

The healing ability of T24 cells was evaluated by cell scratch assays. After detachment with trypsin, T24 cells were placed into 6-well plates at cell concentration of 1 × 10^5^ cells. When cells reached 90% confluence, a 200µL sterile pipette tip was adopted to evenly scratch the plate in a perpendicular direction to produce a uniform and flat wound, followed by 24-h incubation in different groups. Thereafter, the wound healing was respectively observed under a microscope (Olympus) at 0 and 24 h, and the pictures were taken accordingly.

### Western blot

The collected T24 cells were lysed using radio-immunoprecipitation assay solution (SY4680, YITA Biotechnology, Beijing, China), and protein concentration was examined by bicinchoninic acid kits (P0012, Beyotime). Following separating with 10% sodium dodecyl sulfate-polyacrylamide gel electrophoresis, proteins were electrotransferred to polyvinylidene fluoride membranes. Following blockade with 5% skim milk for 2 h, membranes were probed overnight at 4 °C with following rabbit anti-human primary antibodies: matrix metalloproteinase MMP-2 (1/10,000, ab92536, Abcam, Cambridge, UK), light chain 3B (LC3B; 1/50, ab192890), p62 (1/1000, ab207305), phosphorylated p53 (p-p53; 1/1000. ab33889), p53 (1/2000, ab179477), p-mTOR (1/2000, ab109268), mTOR (1/10,000, ab134903), and β-actin (1/1000, ab8227), followed by 1-h incubation with the horseradish peroxidase-labeled goat anti-rabbit secondary antibody IgG (1/50,000, ab205718) at room temperature. Afterward, the images were developed with enhanced chemiluminescence working solution (E412-01, Vazyme). The bands were quantified utilizing Image-Pro Plus 6.0 (Media Cybernetics), with β-actin as an internal reference.

### Statistical analysis

All data were processed and analyzed using SPSS 21.0 statistical software (IBM Corp., Armonk, NY, USA), and graphing was performed using GraphPad Prism 8.0 software (GraphPad Software). The Shapiro-Wilk test was adopted to test the normal distribution of data. The experimentation was duplicated thrice and the results were averaged. Three technical repetitions of cell experiments were conducted, and data were exhibited as mean ± standard deviation (SD). An unpaired independent sample *t*-test was conducted for comparisons between two groups. One-way analysis of variance (ANOVA) was performed for multi-group comparisons, and Tukey’s multiple comparisons test was implemented for post-hoc test. Two-sided tests were used, with *p* < 0.05 indicating statistical significance.

## Results

### Sakuranin suppressed T24 cell proliferation and accelerated apoptosis

**Fig. 1:**
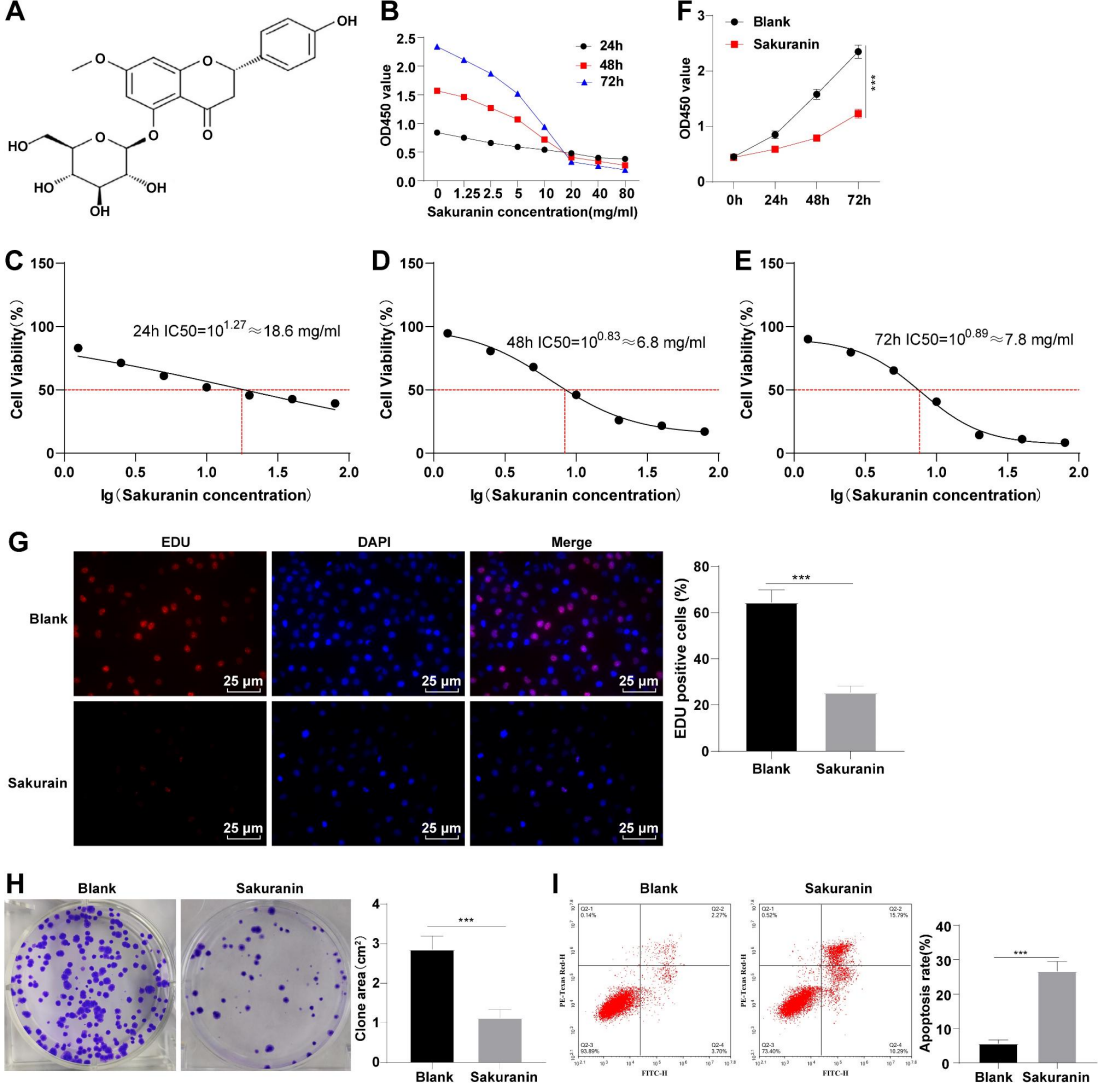
Sakuranin suppressed T24 cell proliferation and accelerated apoptosis. **A:** Molecular structure of Sakuranin; **B:** T24 cells were treated with Sakuranin at concentrations of 0, 1.25, 2.5, 5.0, 10, 20, 40, and 80 mg/mL for 24, 48, and 72 h. Cell viability was detected by CCK-8 assay; **C-E:** The 24-, 48- and 72-h IC50 were analyzed by GraphPad Prism 8.0; **F:** Effect of Sakuranin at 48-h IC50 concentration (6.8 mg/mL) on cell viability was assessed by CCK-8 assay; **G:** Cell proliferation was assessed by EdU assay; **H:** Cell colony formation area was examined using plate clone formation assay; **I:** Cell apoptosis was assessed by flow cytometry. Data were expressed as mean ± SD, and three technical repetitions of cell experiments were conducted. Comparisons between two groups were performed by independent *t*-test. ***p < 0.001

The essential role of flavonoids in BC has been extensively studied [12], but the function of Sakuranin (Fig. 1A), a common flavonoid, in BC is unclear. To investigate the exact effect of Sakuranin on T24 cells, T24 cells were firstly treated with different concentrations (0, 1.25, 2.5, 5.0, 10, 20, 40 and 80 mg/mL) of Sakuranin for 24, 48, and 72 h. The changes in cell viability were detected by CCK-8 assay, which revealed that Sakuranin could prevent T24 cell proliferation in a concentration-dependent manner at 24, 48 and 72 h, with the inhibitory effect at 48 and 72 h stronger than at 24 h (Fig. 1B). Through GraphPad Prism 8.0 software, the 24-, 48- and 72-h IC50 were calculated to be approximately 18.6 mg/mL, 6.8 mg/mL and 7.8 mg/mL, respectively (Fig. 1C-E). T24 cells show the highest sensitivity to Sakuranin after 48 h of treatment. Additionally, referring to similar studies [22], the 48-h IC50 concentration was used for subsequent experiments. We next assessed the effect of Sakuranin on T24 cell proliferation by CCK-8 assay, which demonstrated a lowered proliferative ability upon Sakuranin treatment (Fig. 1F, p < 0.001), EdU assay demonstrated that Sakuranin repressed cell proliferation and reduced the percentage of EdU positive cells (Fig. 1G, p < 0.001), and plate clone formation assay also confirmed the inhibitory role of Sakuranin in T24 cell colony formation area (Fig. 1H, all *p* < 0.001). Further, flow cytometry manifested that Sakuranin significantly impelled T24 cell apoptosis (Fig. 1I, p < 0.001). Taken together, Sakuranin could impede proliferation and potentiate apoptosis of T24 cells.

### Sakuranin repressed T24 cell migration and invasion

**Fig. 2:**
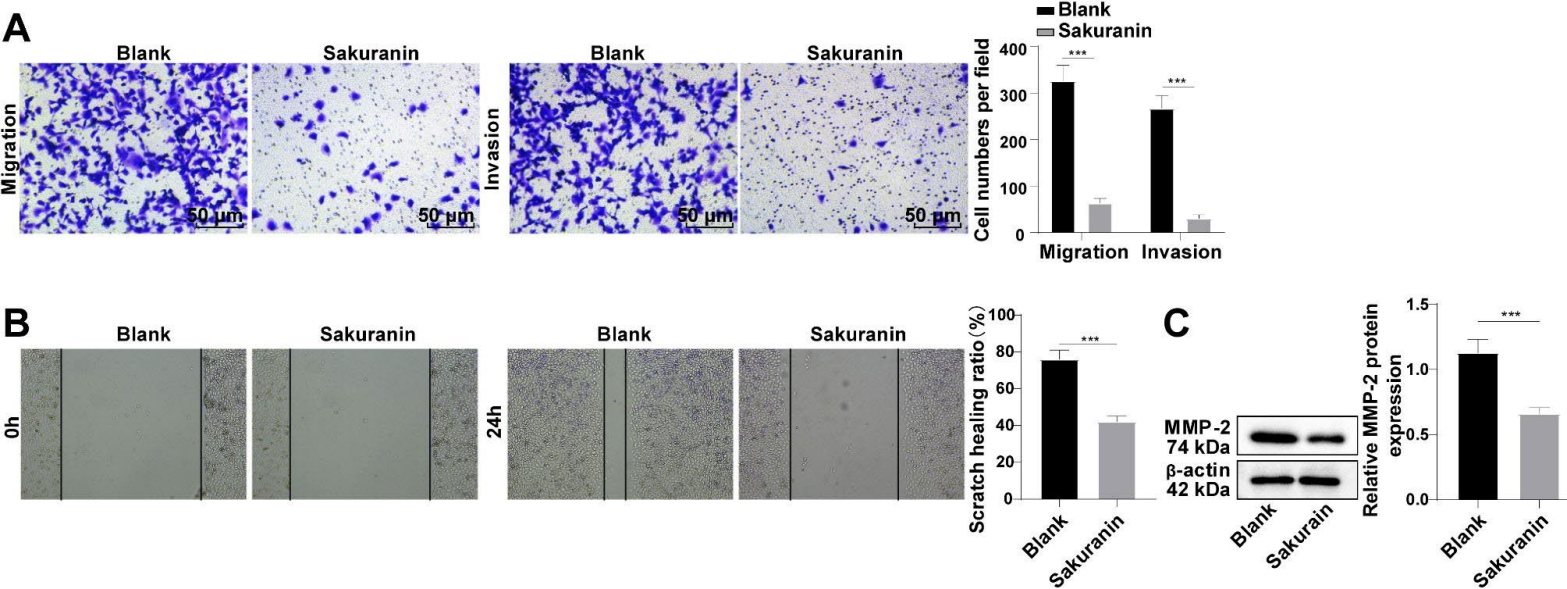
Sakuranin repressed T24 cell migration and invasion. **A:** Transwell assay evaluated cell migration and invasion, and crystal violet staining was used to observe and count cells migrating or invading into the basolateral chamber of the Transwell chambers; **B:** Scratch test assessed wound healing ability of cells; **C:** MMP-2 protein level was assessed by Western blot. Data were presented as mean ± SD, and three technical repetitions of cell experiments were conducted. An independent *t*-test was adopted for comparisons between two groups. ***p < 0.001

Next, the alternations in cell migration and invasion after 48 h of Sakuranin treatment were examined by Transwell assays, which showed that Sakuranin treatment evidently reduced the number of T24 cells migrating and invading into the basolateral chamber of Transwell chambers (Fig. 2A, all *p* < 0.001). As indicated by scratch test, Sakuranin reduced the percentage of scratch healing in T24 cells, and weakened their migration and repair abilities (Fig. 2B, p < 0.001). The further detection of the protein level of MMP-2, a cell invasion and migration-associated protein by Western blot elicited that Sakuranin diminished the protein level of MMP-2 (Fig. 2C, p < 0.001). The above results demonstrated the suppressive roles of Sakuranin in migration and invasion of T24 cells.

### Sakuranin suppressed malignant biological behaviors of T24 cells by triggering autophagy

**Fig. 3:**
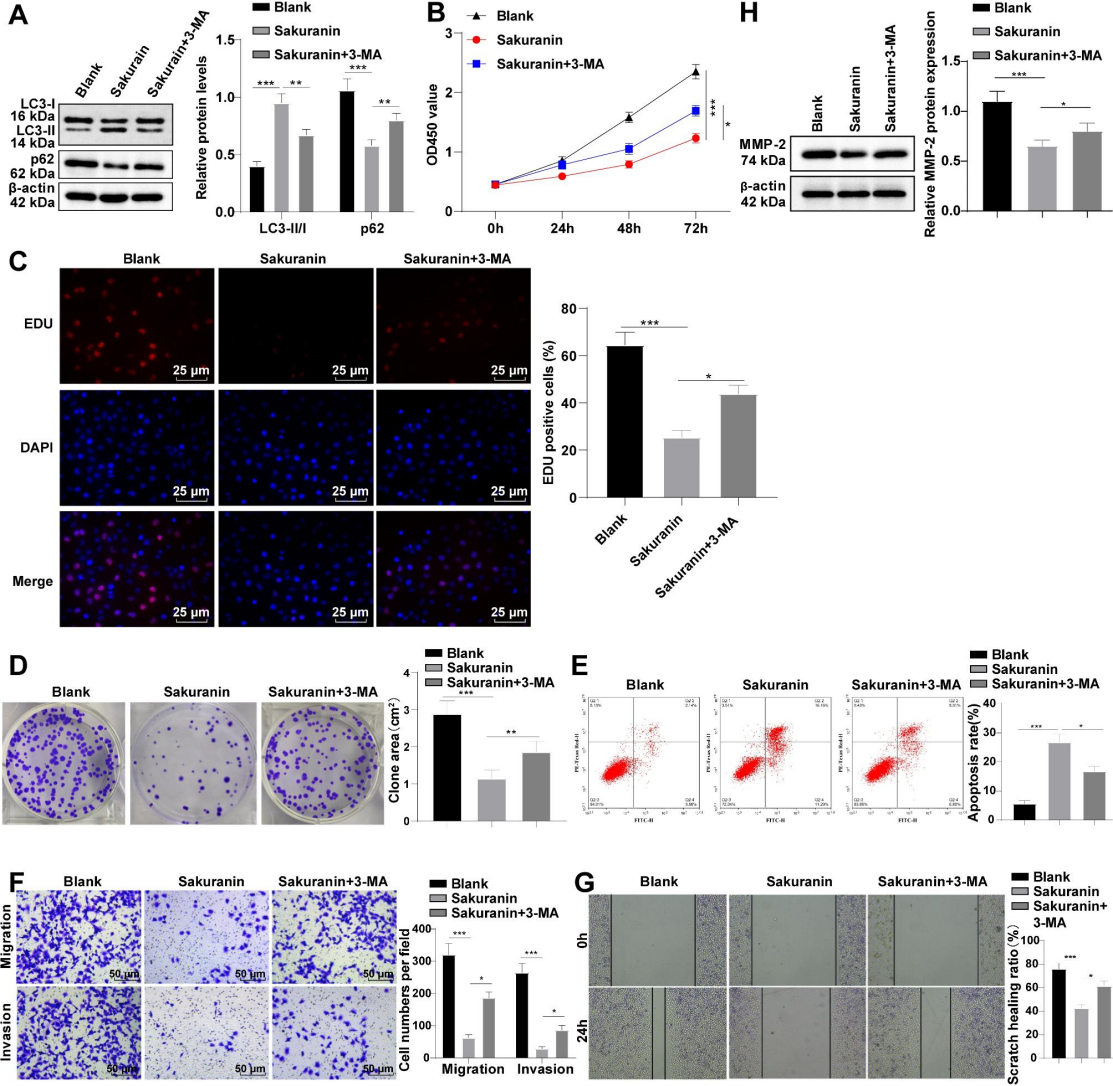
Sakuranin abated malignant biological behaviors of T24 cells by triggering autophagy. **A:** Western blot determined protein levels of autophagy markers (LC3-I, LC3-II, and p62); **B:** CCK-8 assay assessed cell proliferation; **C:** Cell proliferative activity was evaluated by EdU assay; **D:** Plate clone formation assay assessed cell colony formation area; **E:** Flow cytometry evaluated cell apoptosis; **F:** Transwell assay analyzed cell migration and invasion, and crystal violet staining was used to observe and count cells migrating or invading into the basolateral chamber of the Transwell chambers; **G.** Scratch test detected cell wound healing ability; **H:** MMP-2 protein level was assessed by Western blot. Data were exhibited as mean ± SD, and three technical repetitions of cell experiments were conducted. An independent t-test was used for comparisons between two groups, and one-way ANOVA was adopted for multi-group comparisons, followed by Tukey’s test. *p < 0.05, **p < 0.01, ***p < 0.001

Prior research has indicated that the anti-tumor effects of some flavonoids are closely pertinent to cell autophagy [23]. To validate whether the regulatory effect of Sakuranin on T24 cell biological behaviors is associated with autophagy, we determined the protein levels of autophagy markers (LC3-I, LC3-II, and p62) using Western blot. The ratio of LC3-II/I was elevated and p62 protein level was decreased after Sakuranin treatment (Fig. 3A, all *p* < 0.001), which indicated the promotion effect of Sakuranin on T24 cell autophagy. After treatment with a combination of 3-MA (an autophagy inhibitor) with Sakuranin, Western blot revealed a decrease in the LC3-II/I ratio and an increase in p62 level (Fig. 3A, all *p* < 0.01), indicating that T24 cell autophagy was successfully inhibited by 3-MA. Subsequently, CCK-8, EdU and plate clone formation assays were implemented to assess cell proliferation, which elicited that 3-MA treatment promoted T24 cell proliferation, increased EdU positive cell percentage and colony formation area, suggesting that the inhibition of Sakuranin on T24 cell proliferation was partially antagonized by 3-MA (Fig. 3B-D, all *p* < 0.05). Flow cytometry showed a decrease in cell apoptosis after 3-MA treatment (Fig. 3E, p < 0.05). Transwell and scratch assays illustrated that 3-MA increased the number of migrating and invading cells, enhanced their scratch healing ability (Fig. 3F-G, all *p* < 0.05), Western blot demonstrated that MMP-2 protein level was elevated (Fig. 3H, p < 0.05), suggesting that 3-MA partially alleviated the inhibitory effects of Sakuranin on migrative and invasive abilities of T24 cells. Altogether, Sakuranin exerted roles in suppressing malignant biological behaviors of T24 cells via promotion of autophagy.

### Sakuranin activated the p53/mTOR pathway in T24 cells

**Fig. 4:**
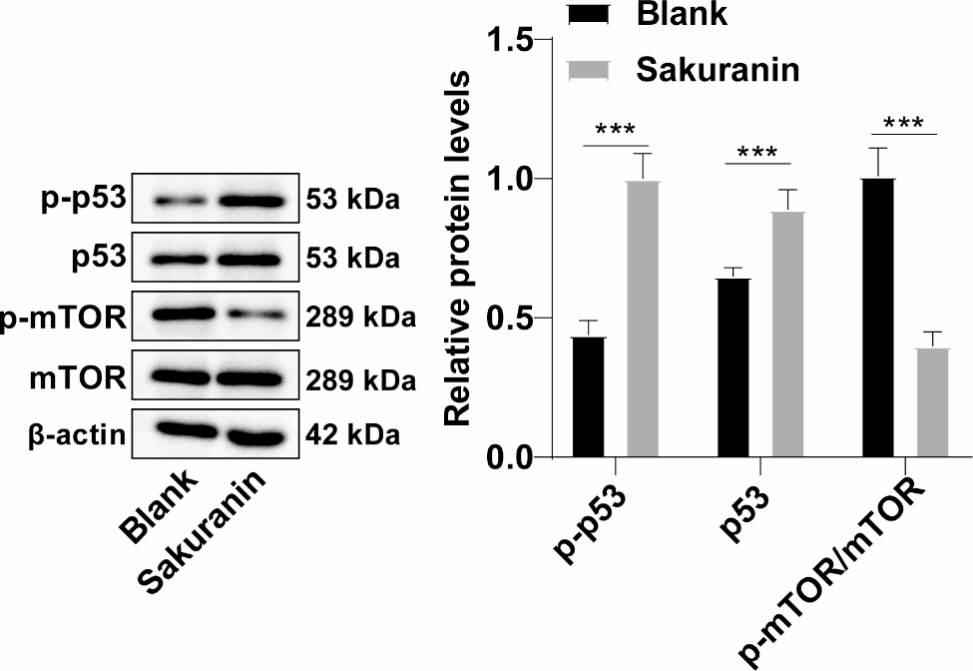
Sakuranin activated the p53/mTOR pathway in T24 cells. Activation of the p53/mTOR signaling pathway was detected by Western blot. Data were displayed as mean ± SD, and three technical repetitions of cell experiments were conducted. Comparisons between two groups were conducted by the independent *t*-test, ***p < 0.001

The p53/mTOR signaling is one of the imperative pathways in controlling cell autophagy [20], and hence we hypothesized that Sakuranin may regulate T24 cell autophagy through the p53/mTOR pathway. Western blot results evinced that both p-p53 and p53 levels were significantly upregulated but the ratio of p-mTOR/mTOR was lowered in T24 cells after Sakuranin treatment (Fig. 4, all *p* < 0.001), which suggested that Sakuranin could regulate the p53/mTOR pathway in T24 cells.

### Sakuranin limited malignant biological behaviors of T24 cells by triggering autophagy through activating the p53/mTOR pathway

**Fig. 5:**
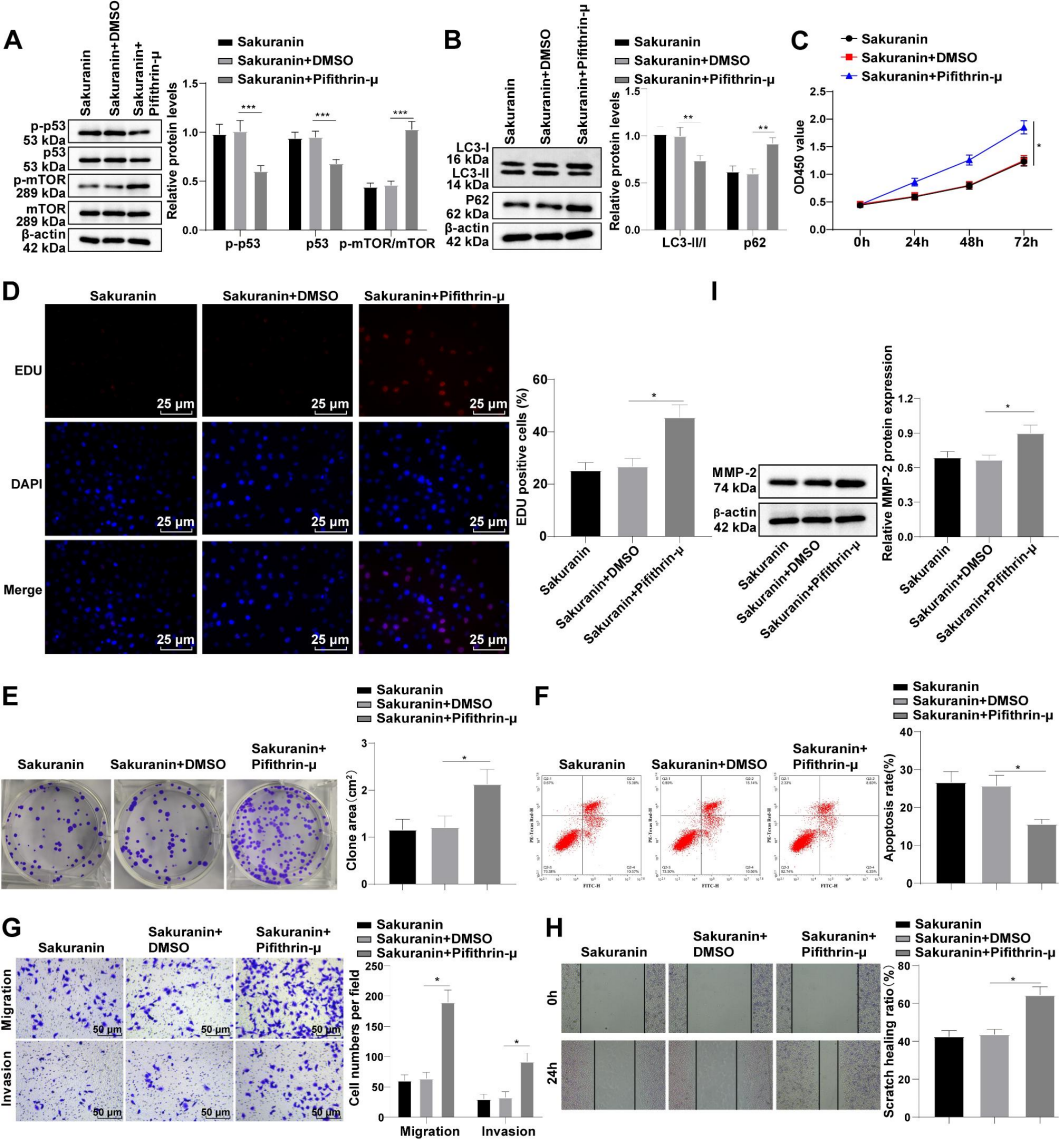
Sakuranin limited malignant biological behaviors of T24 cells by triggering autophagy through activating the p53/mTOR pathway. **A:** Western blot assay detected the activation of the p53/mTOR pathway; **B:** Western blot determined the protein levels of autophagy markers LC3-I, LC3-II, and p62; **C:** CCK-8 assay assessed cell proliferation; **D:** Cell proliferative activity was evaluated by EdU assay; **E:** Plate clone formation assay detected cell colony formation area; **F:** Flow cytometry detected cell apoptosis rate; **G:** Transwell assay assessed cell migration and invasion, and crystal violet staining was used to observe and count cells migrating or invading into the basolateral chamber of the Transwell chambers; **H:** Cell scratch assay evaluated cell wound healing ability, **I:** MMP-2 protein level was assessed by Western blot. Data were presented as mean ± SD, and technical repetitions of cell experiments were implemented thrice. One-way ANOVA was conducted for comparisons among multiple groups, followed by Tukey’s test. *p < 0.05, **p < 0.01, ***p < 0.001

To further elucidate the role of the p53/mTOR pathway in the action of Sakuranin in BC cell biological behaviors, we treated T24 cells with Pifithrin-µ (an inhibitor of p53) in combination with Sakuranin. Western blot analyses revealed significant decreases in p-p53 and p53 levels but an increase in the p-mTOR/mTOR ratio (Fig. 5A, all *p* < 0.001), indicating that Pifithrin-µ successfully impeded activation of the p53/mTOR pathway. The ratio of LC3-II/I was reduced and p62 level was elevated after Pifithrin-µ treatment (Fig. 5B, all *p* < 0.01), suggesting the suppression of Pifithrin-µ on autophagy. Subsequently, through a series of experiments, we found that Pifithrin-µ facilitated cell proliferation, increased colony formation area and EdU positive cell percentage, suggesting that Pifithrin-µ partially restored the proliferative capacity of Sakuranin-treated T24 cells (Fig. 5C-E, all *p* < 0.05), and decreased apoptosis (Fig. 5F, p < 0.05). Additionally, Pifithrin-µ increased the number of invading and migrating cells, scratch healing ability (Fig. 5G-H, all *p* < 0.05) and MMP-2 protein level (Fig. 5I, p < 0.05), suggesting that Pifithrin-µ partially weakened the inhibition of Sakuranin on T24 cell migration and invasion. In summary, Sakuranin inhibited the proliferation, migration, and invasion of T24 cells and stimulated apoptosis by activating the p53/mTOR pathway to stimulate autophagy.

## Discussion

BC ranges from recurrent noninvasive and unaggressive tumors to invasive, aggressive, or advanced-stage tumor that has a high disease-specific mortality and requires potent and multi-modal treatment [24]. Intrinsically, BC shows a distinctive feature of intratumoural and intertumoural heterogeneity at the cellular, transcriptional, and genomic levels, and this heterogeneity ultimately contributes to relapse after therapy and drug resistance, causing poor survival outcomes [25]. Currently, natural flavonoids posse anti-cancerous activities through an array of pathways, and they can trigger cancer cell apoptosis and repress proliferation [10]. In this study, we focused on delving into the potential role and mechanism of Sakuranin (a type of flavonoid) in BC cell behaviors. The results elucidated that Sakuranin prevented proliferation, triggered apoptosis, and impeded invasion and migration of T24 cells by facilitating autophagy via the p53/mTOR pathway.

A prospective study performed in 10 European countries reveals that a higher dietary intake of flavonoids corresponds to a lower risk of BC [26]. Previous researchers have also pointed out that the anti-cancer activity of flavonoids, which can target all phases of carcinogenesis including metastasis, should be implemented into clinical cancer research [27]. Interestingly, the combination of quercetin plus metronomic cyclophosphamide confers anti-fatigue and anti-neoplastic effects with an excellent safety profile in a patient with metastatic BC [28]. Similarly, intraperitoneal injection of kaempferol in BC mice contributes to the reduced tumor weight, increased apoptotic cells, and decreased levels of growth related markers, including c-Fos, c-Met, and cyclin B1 in tumor tissues, thus hampering tumor development [29]. Flavonoids have been shown to engage in arresting the cell cycle, stimulate apoptosis, and curb BC cell proliferation [30]. Specifically, reduction in T24 cell number and viability as well as apoptosis-related biophysical alterations (such as increased aggregation and roughness membrane proteins) are noted following quercetin treatment [31]. Sakuranin belongs to a group of flavanones [13], but there is no reports regarding the effect of Sakuranin on BC. In light of this, our study was conducted to fill the gap and further advance the knowledge in this research area. The obtained finding elicited that Sakuranin suppressed proliferative action in a concentration-dependent mode, accelerated apoptotic, and repressed migrative and invasive abilities of T24 cells.

Autophagy refers to a mechanism through which cellular material is sent to the lysosomes for degradation, causing an essential basal turnover of cell components as well as providing energy and macromolecular precursors [32]. As well-known, autophagy is considered to be a novel and promising target for cancer therapy, which is a contributor to BC cell apoptosis [33]. LC3 and p62 are frequently-used and vital markers of autophagosomes, and an increase in LC3II is indicative of the induction of autophagy, while elevated p62 level is linked with autophagy inhibition [34]. It is interesting to note that Epigallocatechin-3-gallate treatment is able to result in autophagosome formation and significantly enhance autophagy activation in 5,637 and T24 cells by upregulating LC3BII and Beclin [35]. Accordingly, we proposed a hypothesis that Sakuranin may regulate cell biological behaviors through autophagy. In Sakuranin-treated T24 cells, the ratio of LC3-II/I was revealed to be raised, and p62 level was diminished. In our experiments, T24 cells were therefore treated with 3-MA (an autophagy inhibitor) and Sakuranin for further validation. As expected, Sakuranin-induced suppression of T24 cell proliferation, invasion, and migration, and the promotion of apoptosis were partly antagonized by 3-MA treatment, consistent with prior results that blocking autophagy with 3-MA increased Fangchinoline-induced apoptosis in BC cells lines [21]. Altogether, Sakuranin exerted roles in blocking malignant biological behaviors of T24 cells via promotion of autophagy.

p53 is vital in responding to DNA damage, and dysfunctional p53 causes cell malignant transformation [36]. In particular, the p53-mediated mTOR regulation exerts a critical function in tumor suppression [37]. Compelling evidence reveals that galangin-induced apoptosis in BC cells is dependent on the p53 pathway activation [17]. In 5,637 cells, Epigallocatechin-3-gallate treatment significantly upregulates mTOR expression, causing a reduced ratio of p-mTOR/mTOR, thus activating autophagy [35]. Chitooligosaccharide can effective trigger pro-apoptosis autophagy in osteosarcoma cells by affecting the p53/mTOR pathway through significant upregulation of p-p53 and p53 and downregulation of p-mTOR [20]. Subsequently, we probed into the precise function of the p53/mTOR pathway in this regulatory mechanism and observed the elevation in p-p53 and p53 levels and decrease in the p-mTOR/mTOR ratio in Sakuranin-treated T24 cells. Furthermore, treatment with Pifithrin-µ (an inhibitor of p53) partially counteracted the suppressive roles of Sakuranin in malignant behaviors of T24 cells.

In conclusion, this present study underlined for the first time that Sakuranin reduced proliferation, invasion, and migration, and enhanced apoptosis of BC cells by stimulating autophagy through activating the p53/mTOR pathway, providing a new theoretical reference for finding new therapeutic agent and targets for the clinical treatment of BC. However, the results lack in vivo validation, and we shall conduct animal experiments to validate the in vivo anti-tumor effect of Sakuranin in further studies. Furthermore, it is worth investigating the molecular and complex mechanisms of Sakuranin in regulating p53 and other possible mechanisms of Sakuranin in BC cell inhibition. What’s more, other potential mechanisms of Sakuranin regulating the progression of BC, such as the PI3K/AKT pathway [38] and the Wnt/β-catenin pathway [39], play important roles in the proliferation, invasion, migration and stemness maintenance of BC. However, it is unclear whether Sakuranin has a regulatory role through the PI3K/AKT, Wnt/β-catenin and other pathways. The mechanism of Sakuranin regulating the progression of BC may be multi-target. However, we only explored the role of Sakuranin through the p53/mTOR pathway, which was one of the limitations of this study. In the follow-up study, we will further explore other potential mechanisms of Sakuranin regulating the progression of BC.

### Electronic supplementary material

Below is the link to the electronic supplementary material.

Supplementary Material 1

Supplementary Material 2

Supplementary Material 3

Supplementary Material 4

Supplementary Material 5

Supplementary Material 6

## Data Availability

All data generated or analysed during this study are included in this article. Further enquiries can be directed to the corresponding author.
