# Supplementary figures and images for: Sakuranin represses the malignant biological behaviors of human bladder cancer cells by triggering autophagy via activating the p53/mTOR pathway
Source: BMC Urol. 2023 Oct 24;23:170. doi: 10.1186/s12894-023-01334-2 (PMC10594733; doi:10.1186/s12894-023-01334-2)

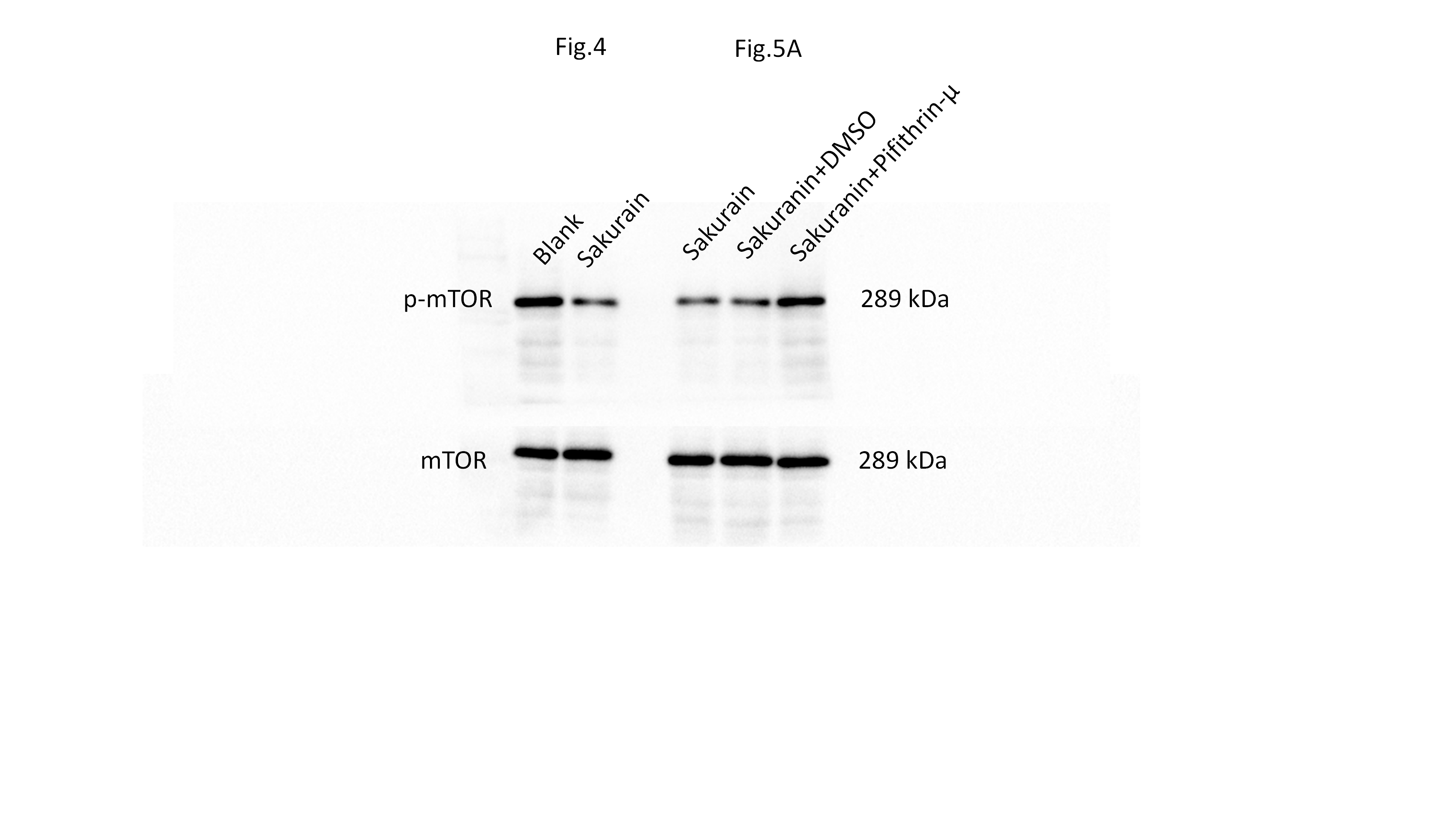

Supplement: Supplementary file 1 — Supplementary Material 1 [file 12894_2023_1334_MOESM1_ESM.png]

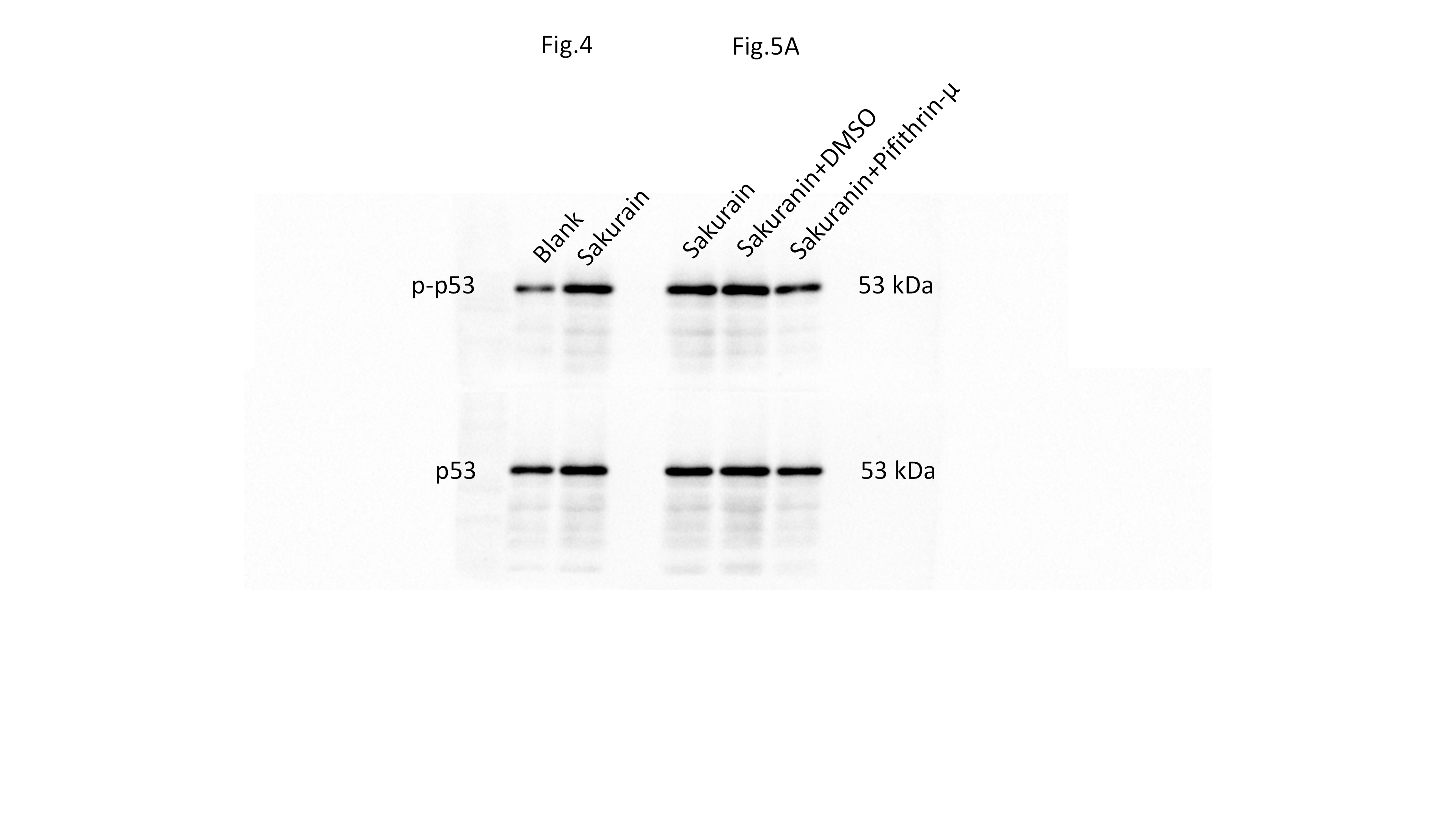

Supplement: Supplementary file 2 — Supplementary Material 2 [file 12894_2023_1334_MOESM2_ESM.png]

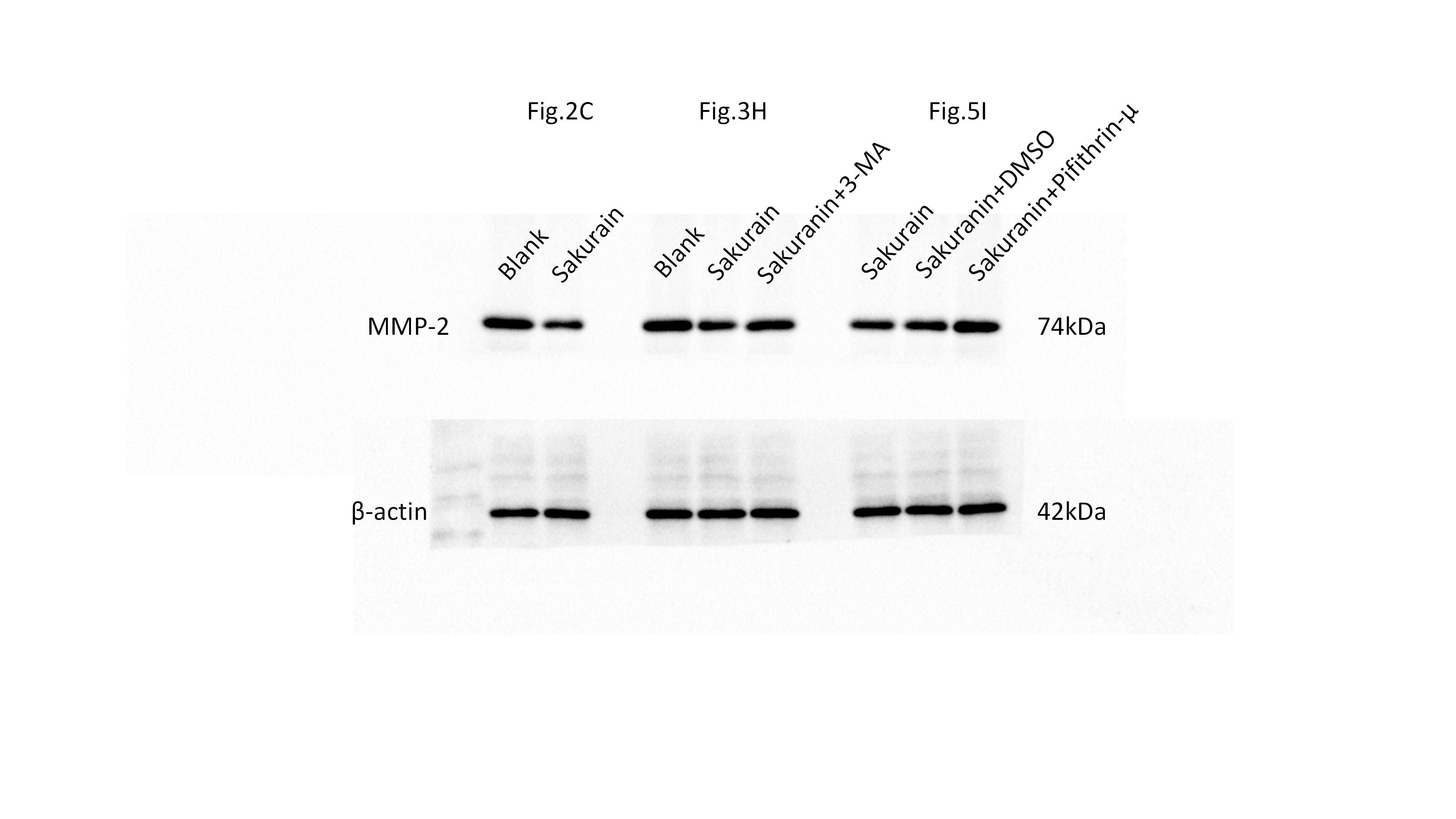

Supplement: Supplementary file 3 — Supplementary Material 3 [file 12894_2023_1334_MOESM3_ESM.png]

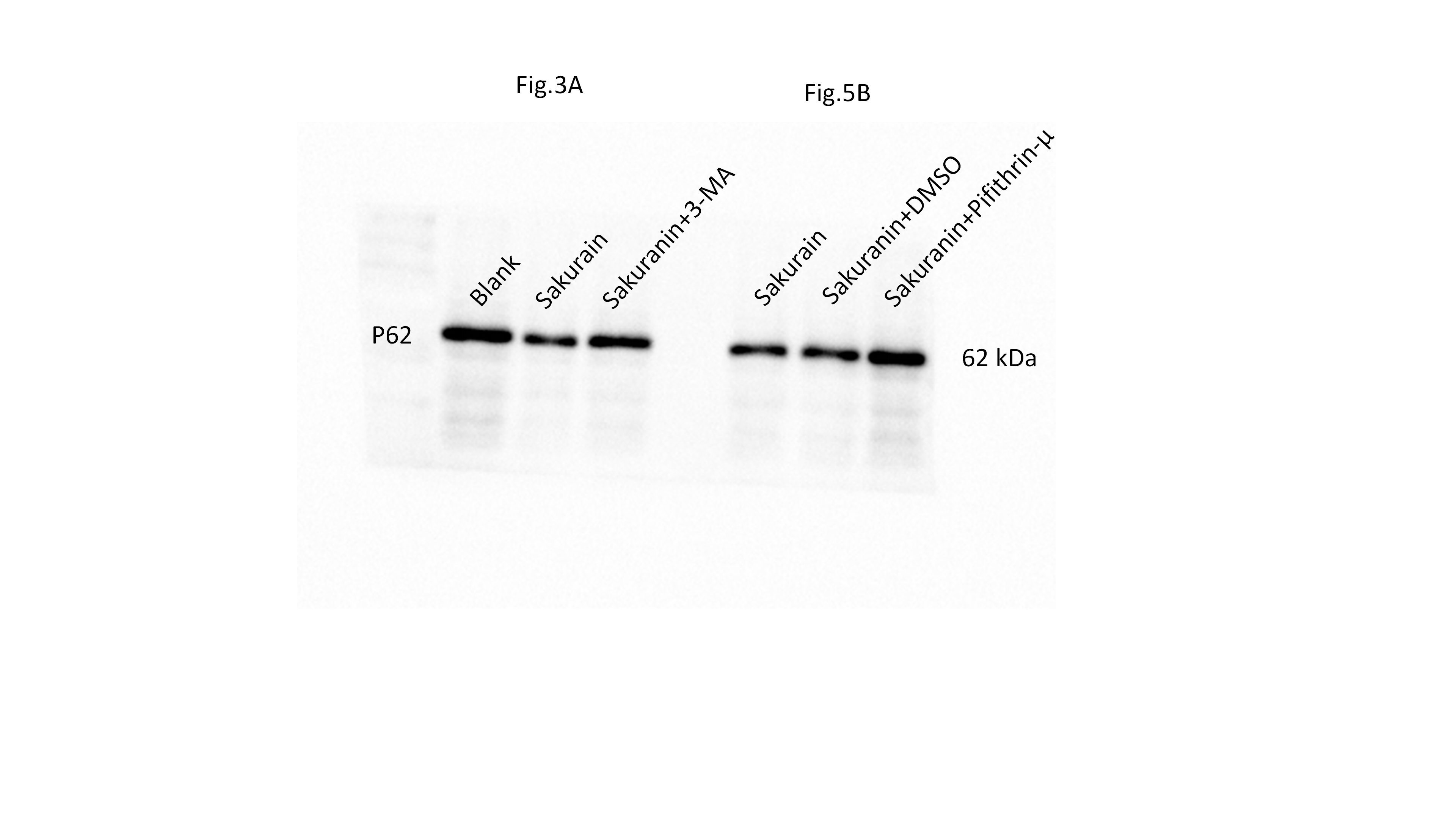

Supplement: Supplementary file 4 — Supplementary Material 4 [file 12894_2023_1334_MOESM4_ESM.png]

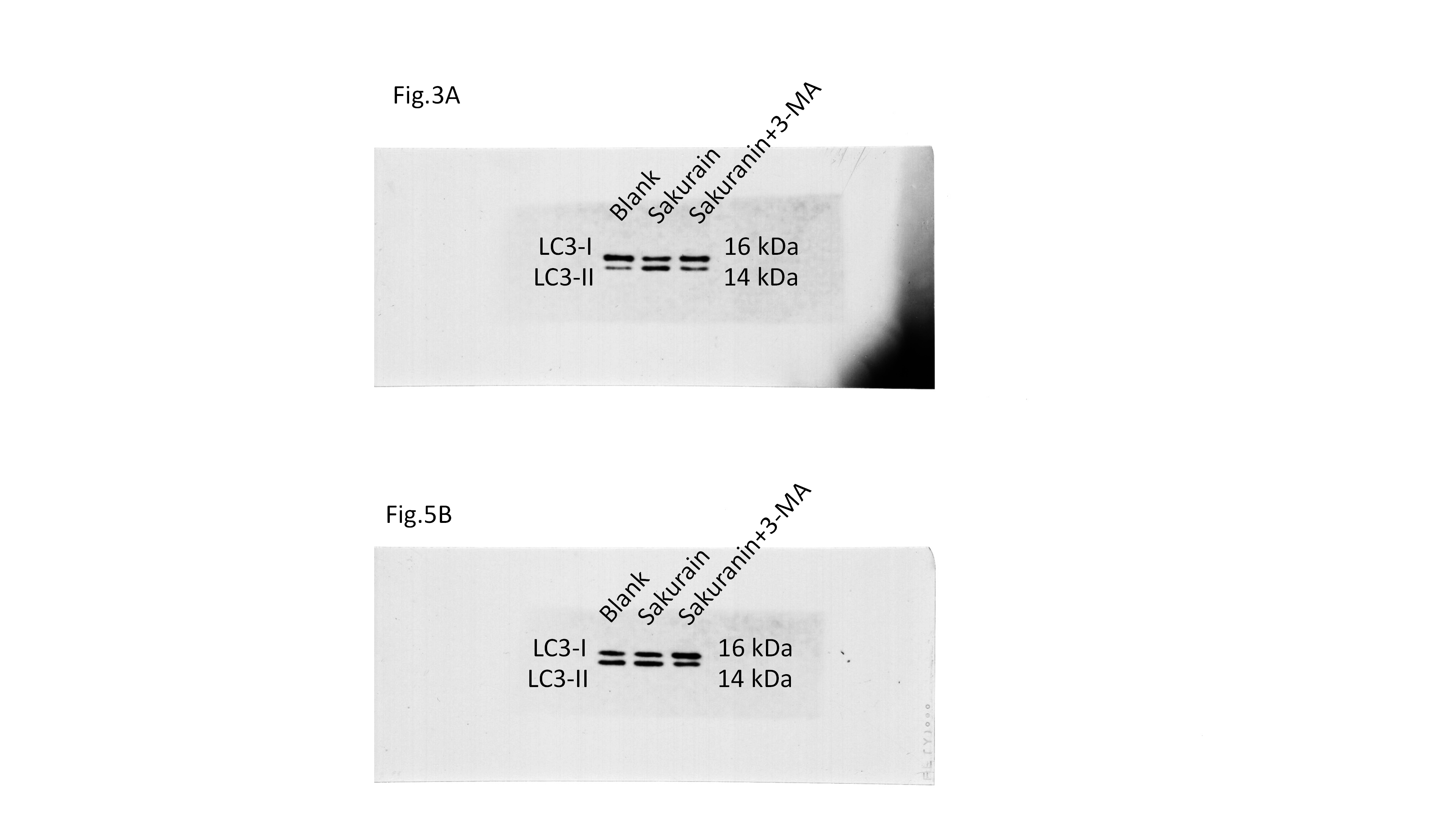

Supplement: Supplementary file 5 — Supplementary Material 5 [file 12894_2023_1334_MOESM5_ESM.png]

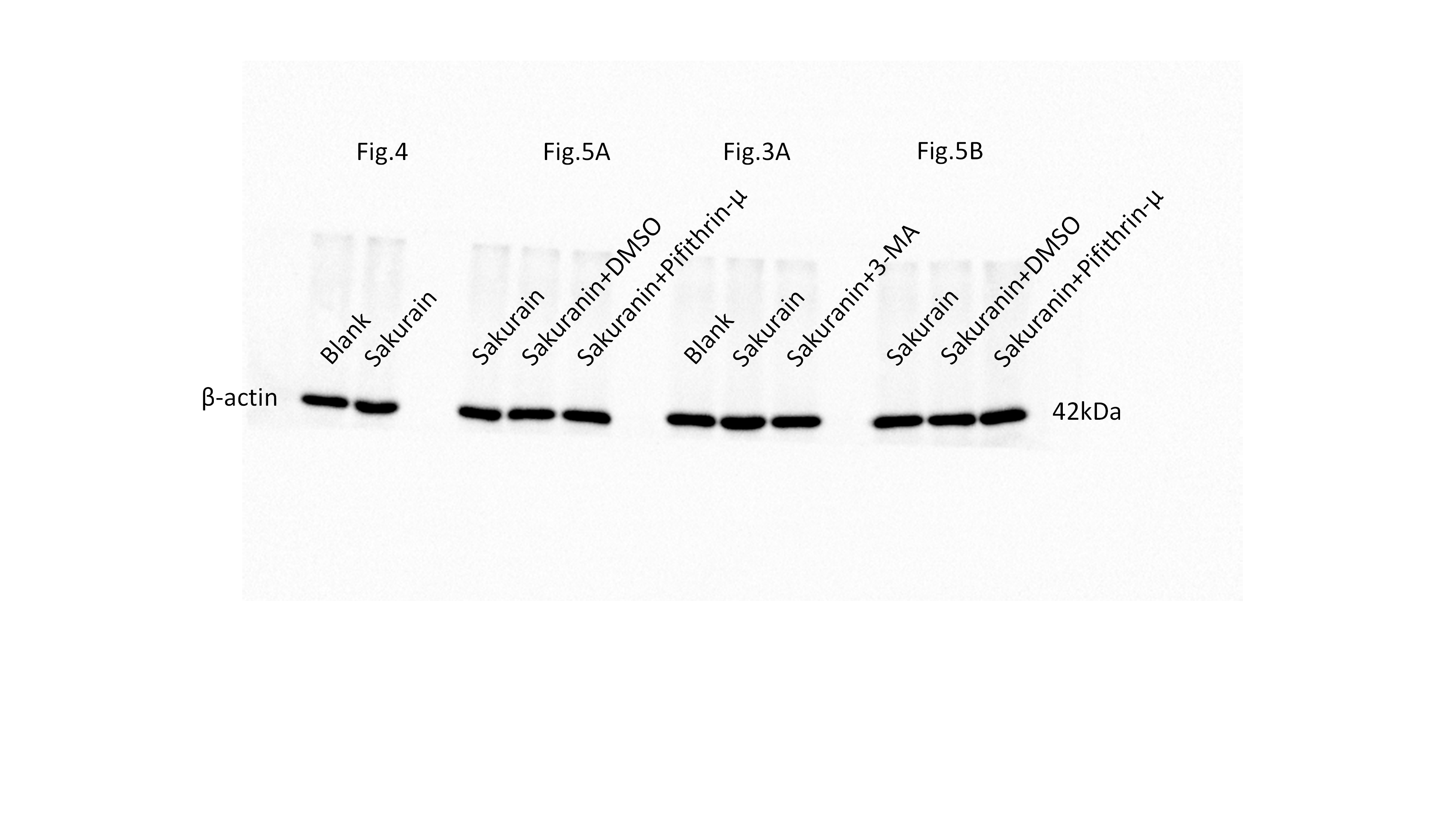

Supplement: Supplementary file 6 — Supplementary Material 6 [file 12894_2023_1334_MOESM6_ESM.png]
